# Modified dispersion relations and a potential explanation of the EDGES anomaly

**DOI:** 10.1140/epjc/s10052-022-10680-8

**Published:** 2022-08-18

**Authors:** Saurya Das, Mitja Fridman, Gaetano Lambiase, Antonio Stabile, Elias C. Vagenas

**Affiliations:** 1 Theoretical Physics Group and Quantum Alberta, Department of Physics and Astronomy, University of Lethbridge, 4401 University Drive, Lethbridge, AB T1K 3M4 Canada; 2 Dipartimento di Fisica E.R: Caianiello, Universita di Salerno, Via Giovanni Paolo II, 132, Fisciano, 84084 Salerno, Italy; 3 INFN-Gruppo Collegato di Salerno, Salerno, Italy; 4 Theoretical Physics Group, Department of Physics, Kuwait University, P.O. Box 5969, Safat, 13060 Kuwait

## Abstract

The *Experiment to Detect the Global Epoch of Reionisation Signature* (EDGES) collaboration has recently reported an important result related to the absorption signal in the Cosmic Microwave Background radiation spectrum. This signal corresponds to the red-shifted 21-cm line at $z \simeq 17.2$, whose amplitude is about twice the expected value. This represents a deviation of approximately $3.8\sigma$ from the predictions of the standard model of cosmology, i.e. the $\Lambda$CDM model. This opens a window for testing new physics beyond both the standard model of particle physics and the $\Lambda$CDM model. In this work, we explore the possibility of explaining the EDGES anomaly in terms of modified dispersion relations. The latter are typically induced in unified theories and theories of quantum gravity, such as String/M-theories and Loop Quantum Gravity. These modified dispersion relations affect the density of states per unit volume and thus the thermal spectrum of the Cosmic Microwave Background photons. The temperature of the 21-cm brightness temperature is modified accordingly giving a potential explanation of the EDGES anomaly.

## Introduction

Predictions of General Relativity (GR) have been tested with high accuracy ranging from the solar system to the cosmological scales. Despite this success, GR is an incomplete theory at short distance and time scales (for example, near black holes and cosmological singularities), and perhaps at large distances as well, where dark components and/or modifications of GR are invoked to explain the accelerated phase of the present Universe. It is expected that the inconsistencies at small scales can be resolved within the framework of quantum gravity (QG), which incorporates the principles of GR and quantum theory, and provides a description of the microstructure of space-time at the Planck scale.

Among the various attempts towards formulating a theory of QG, String/M-theory and Loop Quantum Gravity (LQG) remain as important candidates. A consequence of these theories is that space-time appears non-commutative (NC) at the fundamental level [1–5], and in some situations, may also exhibit a varying speed of light [6, 7]. This gives rise to non-local field theories and a modification of the dispersion relation of the quantum fields in a NC space-time. For example, one of the consequences of String Theory (as well as of M-Theory) is space-time non-commutativity [1], with the latter leading to modified dispersion relations [8]. Related to this is the fact that owing to quantum fluctuations, the usual canonical commutation relation also gets modified $[x,p]=i\hbar (1+\beta p^{2})$ [9–14] (see also Refs. [15–18]). It must be pointed out however, that there are several other approaches to QG that also predict the existence of a minimum measurable length, which in turn represents a natural cutoff and induces a departure from the relativistic dispersion relation. These approaches include space-time foam models [19–21], spin-network in LQG [22], space-time discreteness [23], spontaneous symmetry breaking of Lorentz invariance in string field theory [24] or in NC geometry [25], Horava’s approach [26, 27], and Doubly Special Relativity (DSR) [21, 28, 29]. In Ref. [30], the authors proposed an extension of DSR to include curvature, also known as Doubly General Relativity, in such a way that the geometry of space-time does depend on the energy *E* of the particle used to probe it (*gravity’s rainbow*) [21]. The general form of the modified dispersion relation (MDR) reads [31]

1 $$\begin{aligned} {{E}^{2}}f{{\left( E/{{E}_{P}} \right) }^{2}}-{{p}^{2}}\,c^2\,g{{\left( E/{{E}_{P}} \right) }^{2}}=m^{2}c^4, \end{aligned}$$

where the (rainbow) functions $f(E/E_P)$ and $g(E/E_P)$ depend on the Planck energy $E_P=1.221\times 10^{19}\,\text {GeV}$ (for details see for instance Refs. [32–39]). Now, whenever $f,g \ne 1$, i.e., one deviates from the standard relativistic dispersion relation, as we shall show below, the Planck radiation spectrum changes as well. This in turn may be able to explain the anomaly, which the *Experiment to Detect the Global Epoch of Reionisation Signature* (EDGES) collaboration has recently reported [40]. In the range $z=15-20$, the EDGES collaboration found an anomalous absorption profile, with a brightness temperature minimum at $z_{\mathrm{E}} \simeq 17.2$, which has a magnitude of about a factor of two greater than predicted by the $\Lambda$CDM model. It is this anomaly that we propose to explain using MDRs in this work. It turns out that the standard MDRs do not adequately explain the EDGES anomaly. However, by imposing redshift dependent MDR parameters, or by imposing a non-trivial power dependence for the MDRs, we are able to provide a viable explanation for the EDGES anomaly. A non-trivial power dependence of a MDR is also discussed in Ref. [41].

The rest of the paper is organized as follows. In the next section, we briefly review some of the important special cases of the above MDR. Following this, in Sect. 3, we estimate the parameters in the models that we consider from the results of the EDGES experiment. Finally, we summarize our results and conclude in Sect. 4.

## MDR and modification of thermal spectrum

As stated in the Introduction, MDR is predicted by various theories of QG, and has the most general form of Eq. (1). The rainbow functions can in the most general case be expressed in a power series expansion (MacLaurin series) as $f\left( E/{{E}_{P}} \right) =\sum _{n=0}^\infty \frac{f^{(n)}(0)}{n!}\left( E/E_P\right) ^n$ and $g\left( E/{{E}_P} \right) =\sum _{n=0}^\infty \frac{g^{(n)}(0)}{n!}\left( E/E_P\right) ^n$, where constraints $f(0)=1$ and $g(0)=1$ must be imposed to obtain the standard relativistic dispersion relation at low energies. Here we consider some of the interesting special cases.

- Case 1: $f\left( E/{{E}_{P}} \right) =1$, $g\left( E/{{E}_{P}} \right) =\sqrt{1-\eta \, (E/E_P)^\omega }$, which is one of the most studied in literature. Here $\eta$ is a parameter which signifies the effective scale of the modification, and $\omega$ is the order of the modification. A complete theory of QG should fix both of them. However, in this work we study the modifications for different values of $\eta$ and $\omega$, and in particular, we consider three special cases. The first case is compatible with LQG and NC space-time [42, 43], while the next two are compatible with the linear and quadratic Generalized Uncertainty Principle (GUP) respectively [44, 45]:
  i) $$\omega =1$$ and $$\eta >0$$ $$\Longrightarrow $$ $$f\left( E/{{E}_{P}} \right) =1$$, $$g\left( E/{{E}_{P}} \right) =\sqrt{1-\eta \, E/E_P}~,$$
  ii) $$\omega =1$$ and $$\eta =\mp 2\alpha _0$$ $$\Longrightarrow $$ $$f\left( E/{{E}_{P}} \right) =1$$, $$g\left( E/{{E}_{P}} \right) =\sqrt{1\pm 2\alpha _0\, E/E_P}~,$$
  iii) $$\omega =2$$ and $$\eta =2\beta _0$$ $$\Longrightarrow $$ $$f\left( E/{{E}_{P}} \right) =1$$, $$g\left( E/{{E}_{P}} \right) =\sqrt{1-2\beta _0\, (E/E_P)^2}~,$$
  where restrictions on $\alpha _0$ from Ref. [44] have been relaxed to include both positive and negative values. In general, $f\left( E/{{E}_{P}} \right) \ne 1$ and, specifically, in the presence of a strong gravitational field $f\left( E/{{E}_{P}} \right) =1/\sqrt{-g_{00}}$, where $g_{00}$ is the 00 component of the metric [44, 45]. However, in the dark ages, most of the hydrogen gas was in a very weak field, and, therefore, we can set $f\left( E/{{E}_{P}} \right) =1$, as far as space-time curvature corrections to the MDR are concerned.
- Case 2: $f\left( E/{{E}_{P}} \right) =\frac{{{e}^{\alpha E/E_P}}-1}{\alpha E/{{E}_{P}}},$ $g\left( E/{{E}_{P}} \right) =1$, proposed for explaining the spectra from GRBs at cosmological distances [19].
- Case 3: $f\left( E/{{E}_{P}}\right) =1$, $g\left( E/{{E}_{P}}\right) =[1+(\lambda E)^\gamma ]^\delta =[1+\lambda '\, (E/E_P)^\gamma ]^\delta$, with $\lambda '=(\lambda E_P)^\gamma$. The case $\delta =1$ has been proposed for models in which a varying speed of light occurs [7]. The case $\gamma =\delta =1$ has been proposed in Refs. [21, 46]. For $\gamma =1$, $\delta =1/2$, and $\lambda =-\eta$, we recover Case 1. GUP provides another case for this form of $g\left( E/{{E}_{P}}\right)$ [45]:
  i) $$\delta =1$$, $$\gamma =1$$ and $$\lambda '=\pm \alpha _0$$ $$\Longrightarrow $$ $$f\left( E/{{E}_{P}} \right) =1$$, $$g\left( E/{{E}_{P}}\right) =1\pm \alpha _0\, E/E_P$$ .

Here $\eta , \alpha _0, \beta _0, \alpha , \lambda '$ are dimensionless parameters, with $\alpha _0$ and $\beta _0$ to be the linear and quadratic GUP parameters, respectively. It is often assumed that $\eta , \alpha _0, \beta _0, \alpha ,\lambda '\sim \mathcal{O}(1)$, so that the modifications of the dispersion relations are non-negligible at Planck scales. However, one may relax such a restriction and investigate signals of new physics at a new intermediate scale $\lambda _{new}\sim {\eta }{\ell _{P}} \sim {\alpha _0}{\ell _{P}} \sim {\sqrt{\beta _0}}{\ell _{P}} \sim {\alpha }{\ell _{P}} \sim {\lambda '}{\ell _{P}}$. Such a length (energy) scale $\lambda _{new}$ cannot exceed the well-tested electroweak length scale, $\lambda _{EW}\sim {10^{17}}{\ell _{P}}$, so the consequent upper bound $\eta \sim \alpha _0 \sim \sqrt{\beta _0}\sim \alpha \sim \lambda ' \le 10^{17}$.

The MDR given by Eq. (1), for the case of photons reads

2 $$\begin{aligned} E^2 - p^2 c^2 F^2 =0\,, \quad \text {with} \,\, F=\frac{g}{f}\,, \end{aligned}$$

so that, following Refs. [7, 46], one may derive the modified thermal spectrum $\rho _{MDR}$.

The density of states per volume for photons (which have 2 polarization states) is written as

3 $$\begin{aligned} \Omega (p)=\frac{p^{2}}{\pi ^{2}\hbar ^{3}}. \end{aligned}$$

By considering the MDR in Eq. (2) and using $\Omega (E)\,\mathrm {d}E=\Omega (p)\,\mathrm {d}p$, we obtain the following density of states

4 $$\begin{aligned} \Omega (E)=\frac{E^{2}}{\pi ^{2}{\hbar ^{3}}{\hat{c}}^{2}\tilde{c}}, \end{aligned}$$

where the two ‘speeds’ in the above equation turn out to be

5 $$\begin{aligned} {\hat{c}}=\frac{E}{p}=cF\,\,\,\,\,\,\mathrm {and}\,\,\,\,\,\,\tilde{c} =\frac{\mathrm {d}E}{\mathrm {d}p}=\frac{cF}{1-\frac{F'E}{F}}~, \end{aligned}$$

where $F'=\mathrm {d}F/\mathrm {d}E$. Therefore, we can write the modified density of states as

6 $$\begin{aligned} \Omega (E)=\frac{E^2}{\pi ^2\hbar ^3c^3}\frac{1}{F^3}\left| 1-\frac{F'E}{F}\right| ~. \end{aligned}$$

The modified thermal spectrum is then obtained using^1^$\rho _{MDR}(T,E)=2\pi \hbar E\,n(E)\,\Omega (E)$, where $n(E)=\frac{1}{e^{\beta _T E}-1}$ is the Bose-Einstein distribution, $\beta _T=\frac{1}{k_BT}$ is the inverse temperature and $k_B$ is the Boltzmann constant. The modified thermal spectrum then reads as

7 $$\begin{aligned} \rho _{MDR}(E,T) = \rho (E,T) \frac{1}{F^3}{\left| 1-\frac{F'E}{F}\right| }\equiv \rho (E,T)R~, \end{aligned}$$

where

8 $$\begin{aligned} \rho (E,T)=\frac{2}{\pi \hbar ^2 c^3}\frac{E^3}{e^{\beta _T E}-1} \end{aligned}$$

is the standard thermal distribution of photons and *R* is the correction factor, formally defined in the following section. Note that the standard result from Eq. (8) is obtained from Eq. (7) when the MDR parameters vanish, i.e., $\eta , \alpha _0, \beta _0, \alpha , \lambda '\longrightarrow 0$.

## Experimental bounds

In this section we study the effects of the modified thermal spectrum given by Eq. (7), induced by the MDR given in Eq. (2), on the 21-cm cosmology. Details of 21-cm cosmology are given in Appendix A. This is related to the history of the universe, and represents a new framework for probing fundamental physics [49] (for other models see Refs. [50–59]). In particular, we focus on the recent release of the EDGES collaboration [40] (see also Ref. [60]).

EDGES High and Low band antennas probe the frequency ranges 90–200 MHz and 50–100 MHz, respectively, overall measuring the 21-cm signal within the redshift range $z\in 6 - 27$, corresponding to an age of the Universe $t_U\in (100 \mathrm{Myr} - 1 \mathrm{Gyr})$, i.e., the *dark ages*. This includes the epochs of reionization and cosmic dawn, in which the first astrophysical sources form. At $z_{\mathrm{E}} \simeq 17.2$, the observed magnitude of the absorption line^2^ is about a factor of two greater than the one predicted by the $\Lambda$CDM model. At the redshift of the minimum of the 21-cm line, i.e., $z_{\mathrm{E}} \simeq 17.2$, and frequency of CMB radiation, i.e., $\nu _{21}(z_{\mathrm{E}})\simeq 78\,\mathrm{MHz}$, one has a 21-cm brightness temperature $T_{21}(z_{\mathrm{E}})=-0.5_{-0.5}^{+0.2}\,\mathrm{K}$ ($99\%\,\mathrm{C.L.}$, including estimates of systematic uncertainties). Since at $z=z_E$ one has $(1+\delta _{\mathrm{b}})\,x_{H_I}(z_{\mathrm{E}}) \simeq 1$, Eq. (A5) implies $T_{\gamma }(z_{\mathrm{E}})/T_S(z_{\mathrm{E}}) =15^{+15}_{-5.5}$ [49, 60]. Moreover, in the context of the $\Lambda$CDM model, one also gets

9 $$\begin{aligned} T_{\gamma }(z_{\mathrm{E}}) = T_{CMB}(z_{\mathrm{E}}) = T_{CMB,0}\,(1+z_\mathrm{E}) \simeq 50\,\mathrm{K} \end{aligned}$$

and

10 $$\begin{aligned} T_{\mathrm{gas}}(z_{\mathrm{E}}) \simeq T_{CMB}(z_{\mathrm{dec}}^\mathrm{gas})\,\left( \frac{1+z_{\mathrm{E}}}{1+z_{\mathrm{dec}}^{\mathrm{gas}}}\right) ^2 \simeq 6\,\mathrm{K}~, \end{aligned}$$

where $z_{\mathrm{dec}}^{\mathrm{gas}}\simeq 150$ and $T_{\mathrm{dec}}^{\mathrm{gas}} \simeq 410\,\mathrm{K}$ are the redshift and the temperature at the time when the gas and radiation decouple. Using (A5), one infers $T_{21}(z_{\mathrm{E}}) \gtrsim -0.2\,\mathrm{K}$. Notice that the minimum is saturated for $T_{S}(z_{\mathrm{E}}) = T_{\mathrm{gas}}(z_\mathrm{E})$, which corresponds to $T_{\gamma }(z_{\mathrm{E}})/T_{\mathrm{gas}}(z_\mathrm{E}) \simeq 8$. As a consequence of these results, one finds that the best fit value for $T_{21}(z_E)$ is $\sim 2.5$ times lower than expected within the $\Lambda$CDM.

The 21-cm CMB photons absorbed at $z_E$ fall clearly in the Rayleigh–Jeans tail since $E_{21} \ll k_BT(z_{\mathrm{E}})$, where $E_{21}$ is the hyperfine transition energy of the hydrogen atom. The energy density of the photons, i.e., Eq. (8), evaluated at $T=T_{CMB}$, reads

11 $$\begin{aligned} \rho _{CMB}(E,z) = \frac{2}{\pi \hbar ^2 c^3}\,\frac{E^3}{e^{\beta _{T_{CMB}}\!(z)\,{E}}-1}~, \end{aligned}$$

where $\beta _{T_{CMB}}\!(z)=\frac{1}{k_BT_{CMB}(z)}$. Only photons with energy $E_{21}$ at $z\simeq z_{\mathrm{E}}$ could be absorbed by the neutral hydrogen producing a 21-cm absorption global signal. For explaining the EDGES results, we consider the $\rho _{MDR}$ given by Eq. (7). Therefore, we define the parameter *R* to study the discrepancy from the $\Lambda$CDM model as

12 $$\begin{aligned} R \equiv \frac{\rho _{MDR}(E_{21},z_E)}{\rho _{CMB}(E_{21},z_E)}=\frac{1}{F^3}{\left| 1-\frac{F'E_{21}}{F}\right| }~, \end{aligned}$$

with $\rho _{MDR}$ and $\rho _{CMB}$ defined in Eqs. (7) and (11), respectively. It may appear that such a modification may affect the optical depth $\tau _\nu$ (introduced in Appendix A) and, therefore, the intensity and shape of the 21-cm line profile. However, as shown in Appendix B, such a modification does not affect $\tau _\nu$ in any way. The experimental values from the EDGES experiment can then be explained by imposing (see Ref. [60] for details)

13 $$\begin{aligned} R = 2.15^{+2.15}_{-0.8} \,. \end{aligned}$$

Parameter *R* in Eq. (12) is then only a function of *F*, $F'$ and *E*, since everything else except the relevant correction cancels out. Since we can in general write the rainbow functions *f* and *g* as a power series in $E/E_P$, we can also write the function $F=g/f$ as a power series expansion

14 $$\begin{aligned} F\left( E/{{E}_{P}} \right) =\frac{g\left( E/{{E}_{P}} \right) }{f\left( E/{{E}_{P}} \right) }=\sum _{n=0}^\infty \frac{F^{(n)}(0)}{n!}\, \left( E/E_P\right) ^n~. \end{aligned}$$

Note that $F(0)=1$, which corresponds to the standard $\Lambda$CDM result. The parameter *R* from Eq. (12) for such a general expression reads

15 $$\begin{aligned} R=\frac{\left| 1-\sum _{n=1}^\infty \frac{F^{(n)}(n-1)}{n!}\left( {E}/{E_P}\right) ^{n}\right| }{\left[ \sum _{m=0}^\infty \frac{F^{(m)}}{m!}\left( {E}/{E_P}\right) ^{m}\right] ^4}~. \end{aligned}$$

Either Eq. (12) or Eq. (15) above can be used to estimate *R* for the cases studied here, compare with experimentally measured values and obtain bounds on the various parameters.

**Fig. 1:**
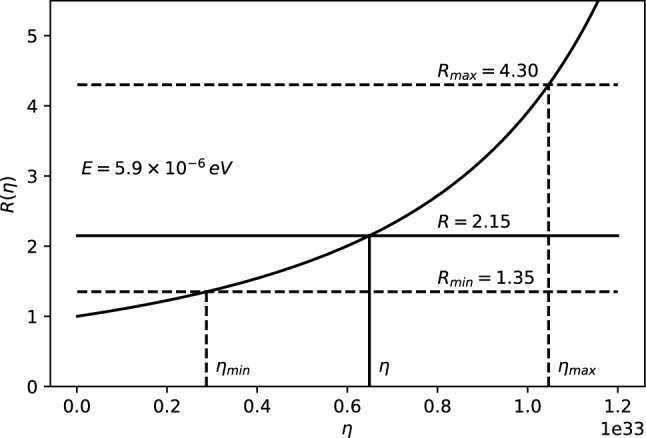
*R* vs $\eta$ for fixed energy $E=E_{12}\simeq 5.9\times 10^{-6}$eV

**Fig. 2:**
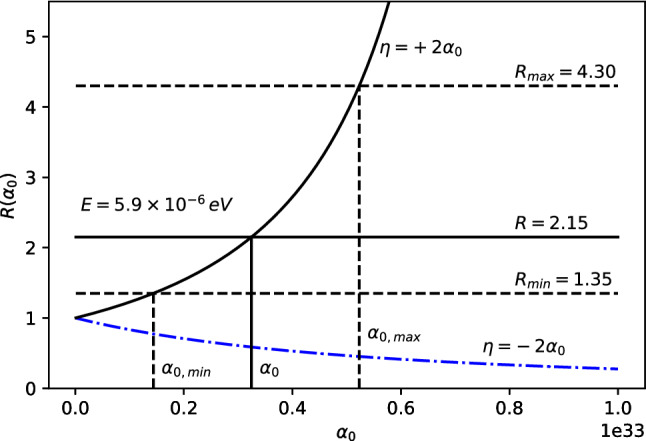
*R* vs $\alpha _0$ for fixed energy $E=E_{12}\simeq 5.9\times 10^{-6}$eV. The $\eta =-2\alpha _0$ branch is presented in dash-dot blue and the $\eta =+2\alpha _0$ branch is presented in solid black

**Fig. 3:**
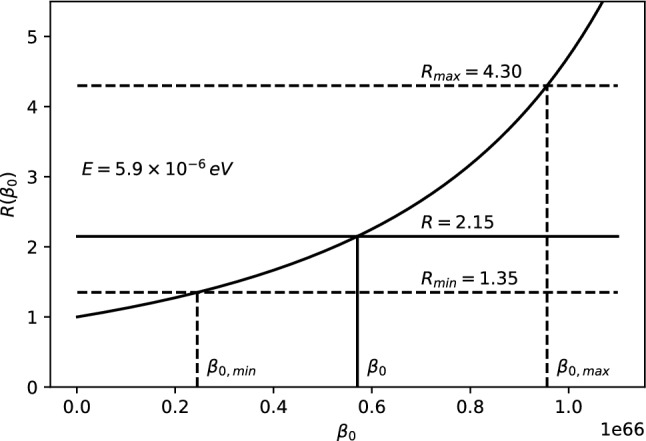
*R* vs $\beta _0$ for fixed energy $E=E_{12}\simeq 5.9\times 10^{-6}$eV

**Fig. 4:**
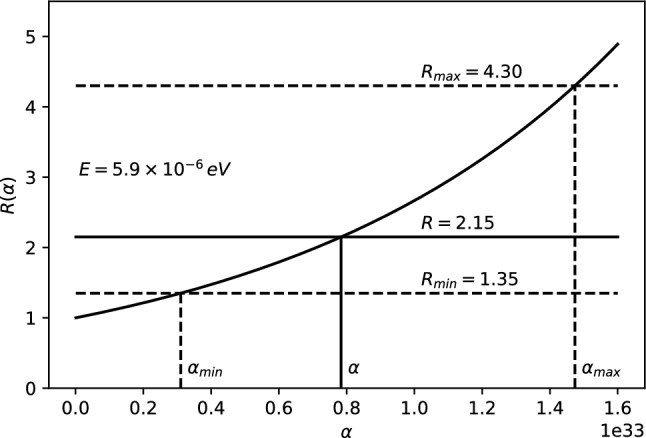
*R* vs $\alpha$ for fixed energy $E=E_{12}\simeq 5.9\times 10^{-6}$eV

**Fig. 5:**
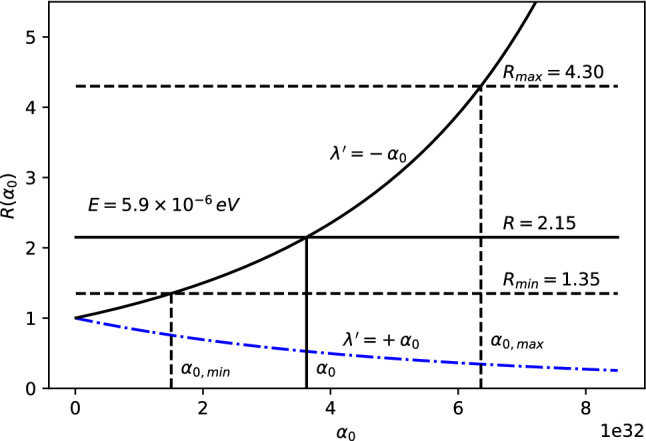
*R* vs $\alpha _0$ for fixed energy $E=E_{12}\simeq 5.9 \times 10^{-6}$eV. The $\lambda '=+\alpha _0$ branch is presented in dash-dot blue and the $\lambda =-\alpha _0$ branch is presented in solid black

- Case 1: $f\left( E/{{E}_{P}} \right) =1$, $g\left( E/{{E}_{P}} \right) =\sqrt{1-\eta \, (E/E_P)^\omega }~$. The ratio *R* reads
  16 $$\begin{aligned} R=\frac{|1-\left( 1-\frac{\omega }{2}\right) \eta \, (E/E_P)^\omega |}{(1-\eta \, (E/E_P)^\omega )^{5/2}}~ \end{aligned}$$
  for arbitrary parameters $\eta$ and $\omega$. We take a look at the special cases:
  i) For $\omega =1$ and $\eta >0$, we have
    17 $$\begin{aligned} R=\frac{|1-\eta \, E/2E_{P}|}{(1-\eta \, E/E_P)^{5/2}}~. \end{aligned}$$
    The ratio *R* is plotted as a function of $\eta$ in Fig. 1. To fit the EDGES experimental bounds, the parameter is fixed at $\eta = 6.5_{-3.6}^{+4.0}\times 10^{32}$.
  ii) For $\omega =1$ and $\eta =\mp 2\alpha _0$ we have
    18 $$\begin{aligned} R =\frac{|1\pm \alpha _0\, E/E_P|}{[1\pm 2\alpha _0\, E/E_P]^{5/2}}~. \end{aligned}$$
    The ratio *R* is plotted as a function of $\alpha _0$ for both branches in Fig. 2. However, only the branch with $\eta =+2\alpha _0$ can fix $\alpha _0$. To fit the EDGES experimental bounds, the parameter is fixed at $\alpha _0=3.2_{-1.8}^{+2.0}\times 10^{32}$.
  iii) For $\omega =2$ and $\eta =2\beta _0$ we have
    19 $$\begin{aligned} R=\frac{1}{(1-2\beta _0\, (E/E_P)^2)^{5/2}}~. \end{aligned}$$
    The ratio *R* is plotted as a function of $\beta _0$ in Fig. 3. To fit the EDGES experimental bounds, the parameter is fixed at $\beta _0=5.7_{-3.3}^{+3.9}\times 10^{65}$.
- Case 2: $f\left( E/{{E}_{P}} \right) =\frac{{{e}^{\alpha E/Ep}}-1}{\alpha E/{{E}_{P}}}$, $g\left( E/{{E}_{P}} \right) =1~$. The ratio *R* reads
  20 $$\begin{aligned} R=\frac{e^{\alpha E/E_{P}} (e^{\alpha E/E_{P}} -1)^2}{(\alpha E/E_P)^2 }\,. \end{aligned}$$
  The ratio *R* is plotted as a function of $\alpha$ in Fig. 4. To fit the EDGES experimental bounds, the parameter is fixed at $\alpha =7.8_{-4.7}^{+6.9}\times 10^{32}$.
- Case 3: $f=1$, $g=\left[ 1+\lambda '\, (E/E_P)^\gamma \right] ^\delta$. The ratio *R* reads
  21 $$\begin{aligned} R=\frac{|1+(1-\delta \gamma )\lambda '\, (E/E_P)^\gamma |}{[1+\lambda '\, (E/E_P)^\gamma ]^{3\delta +1}}~, \end{aligned}$$
  for arbitrary parameters $\lambda '$, $\gamma$ and $\delta$. We take a look at the special case:
  i) For $\delta =1$, $\gamma =1$ and $\lambda '=\pm \alpha _0~$, we have
    22 $$\begin{aligned} R=\frac{1}{(1\pm \alpha _0E/E_P)^4}~. \end{aligned}$$
    The ratio *R* is plotted as a function of $\alpha _0$ for both branches in Fig. 5. However, only the branch with $\lambda '=-\alpha _0$ can fix $\alpha _0$. To fit the EDGES experimental bounds, the parameter is fixed at $\alpha _0=3.6_{-2.1}^{+2.7}\times 10^{32}$.

At this point it should be stressed that the above plots indicate that the MDRs provided by cases 1, 2 and 3, give $(\eta , \alpha _0, \sqrt{\beta _0}, \alpha , \lambda ')\left| _{z=z_E} \approx 10^{32}\right.$ at redshift $z=z_E$. These values are much larger than the bound set by the electroweak scale $\lambda _{EW}/\ell _P\lesssim 10^{17}$. To verify the compatibility with known observations and obtain the bounds on the above parameters in the current epoch ($z=0$), we compare the experimental precision of the CMB temperature $\left( \frac{\delta T}{T}\right) _{exp}=2\times 10^{-4}$ [61] (see also Refs. [62, 63]) of a perfect black body to the theoretical deviation due to MDRs in the current epoch

23 $$\begin{aligned} \frac{\delta T}{T}(z=0)= & {} (R(E)-1)\nonumber \\&\frac{\cosh {\left( \beta _{T_{CMB}}\!(0)\,E\right) } -1}{e^{\beta _{T_{CMB}}\!(0)\,E}-1}\,\frac{2}{\beta _{T_{CMB}}\!(0)\,E}~. \end{aligned}$$

In the above, *R*(*E*) is given by Eq. (12) and $\beta _{T_{CMB}}\!(0)$ is given in terms of the CMB temperature in the current epoch. We obtain Eq. (23) by expressing $\frac{\delta T}{T}$ from $\rho _{MDR}(E,T)=\rho (E,T) R\approx \rho (E,T)+\frac{\mathrm {d}\rho }{\mathrm {d}T}(E,T)\,\delta T$. The parameters in the current epoch then must satisfy an upper bound of $(\eta , \alpha _0, \sqrt{\beta _0}, \alpha , \lambda ')\left| _{z=0} < 10^{28}\right.$ to be consistent with the observed CMB spectrum in the current epoch. The bound obtained from the electroweak experiments is stronger than that, so it should be used as the relevant MDR parameter bound in the current epoch. The EDGES anomaly at $z=z_E$ combined with the above bound at $z=0$ suggest that the above parameters should be increasing functions of the redshift *z*. Therefore, we also expect *R* to increase with *z* for a given energy *E* and have a value of $R\approx 1$ at $z=0$.

The compatibility of such MDRs with epochs earlier than $z_E$ should be taken into consideration as well. For example, in the epoch of the Big Bang Nucleosynthesis (BBN), at $z\approx 3\times 10^8$ [64], a bound of $\beta _0\lesssim 10^{87}$ was obtained in [65] for the quadratic GUP parameter $\beta _0$, which corresponds to an upper bound $\lesssim 10^{44}$ for the MDR parameters. Therefore, the values of the MDR parameters, measured by the EDGES anomaly are consistent with the BBN measurements, even if they increase to $\sim 10^{44}$ at $z\approx 3\times 10^{8}$. This supports the increasing trend of the redshift dependence of the MDR parameters and may in fact provide a clue in determining the exact form of this dependence. Estimations of the MDR parameters from the modified CMB spectrum would not be relevant in the BBN epoch, since it has not been created until the epoch of recombination at $z=1090$ [64].

The standard MDRs used in this work can be found in Refs. [21, 42–46] as mentioned in Sect. 2, but they consider the MDR parameters as constants. The assumption that the MDR parameters are functions of another parameter, such as redshift, is fairly new. However, such an assumption is indirectly supported by Ref. [66], where the author finds a mass/radius dependent GUP parameter. This is also supported by the difference between estimations of the quadratic GUP parameter in tabletop experiments, where $\beta _0>0$ [67–71], and astrophysical/cosmological observations, where $\beta _0<0$ [66, 72–76]. This shows that the MDR parameters can in fact be dependent on scale or redshift.

Since the usual models of modified dispersion relations can not explain the EDGES anomaly, it is also legitimate to investigate if it can be explained by considering the cases analyzed here in which $\eta , \alpha _0, \lambda '=10^{17}$, namely they are fixed to the electroweak scale, while $\omega$ and $\delta$ are treated as free parameters. We only consider cases 1 and 3, since case 2 has no other parameters to tweak. Also, we did not separately consider the special case 1, iii), because it is automatically considered as $\omega \longrightarrow 2$.

**Fig. 6:**
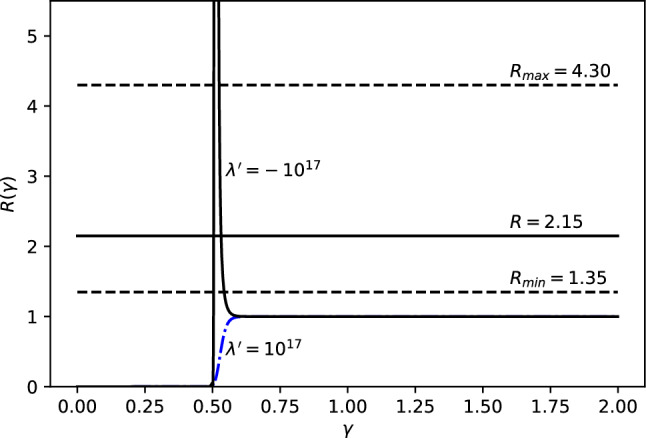
*R* vs $\gamma$ for fixed energy $E=E_{12}\simeq 5.9\times 10^{-6}$eV. The $\lambda '=+10^{17}$ branch is presented in dash-dot blue and the $\lambda '=-10^{17}$ branch is presented in solid black

**Fig. 7:**
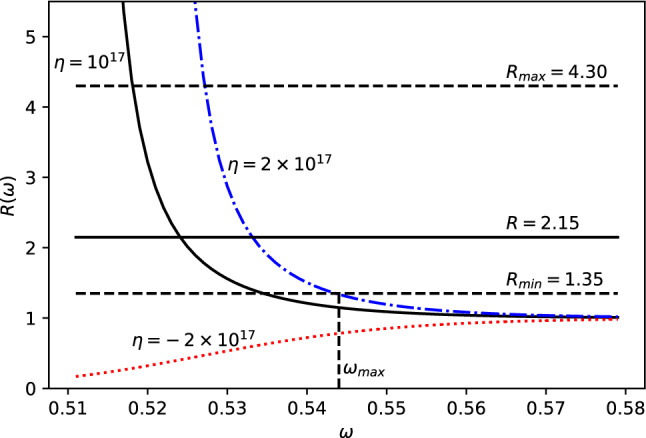
*R* vs $\omega$ for fixed $\eta ,\alpha _0=10^{17}$ and energy $E=E_{12}\simeq 5.9\times 10^{-6}$eV. The solid black, the dash-dot blue and dotted red lines represent cases 1) i) and ii) (positive and negative branch) respectively

**Fig. 8:**
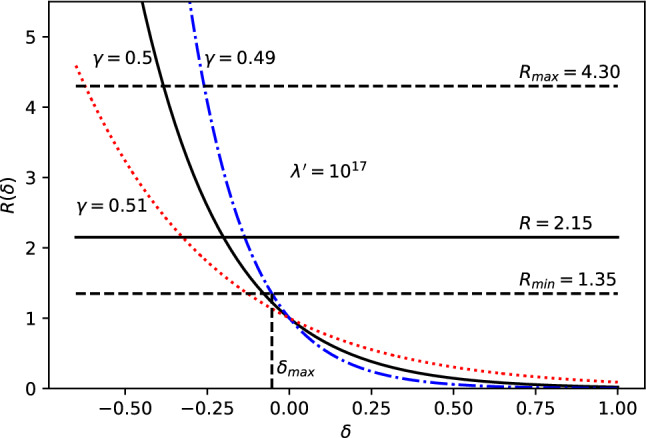
*R* vs $\delta$ for fixed $\lambda '=10^{17}$ and energy $E=E_{12}\simeq 5.9\times 10^{-6}$eV. The dash-dot blue, solid black and dotted red lines represent $\gamma =0.49,\,0.50,\,0,51$ respectively

In Fig. 6, we plot *R* from Eq. (21) vs $\gamma$ for fixed $\lambda ' =\pm 10^{17}$ and for fixed $\delta =1$. The values of *R* for $\lambda '=10^{17}$ fall outside the EDGES bounds and cannot provide an explanation for the EDGES anomaly. However, the values of *R* for $\lambda '=-10^{17}$ fall inside the EDGES bounds twice in a narrow range of $\gamma$ around $\gamma \approx 0.5$ and can therefore provide an explanation for the EDGES anomaly. Changing the $\delta$ parameter only moves the peak to a different location.

At this point a number of comments are in order. First, the power dependencies on $\omega$ and $\delta$ of these cases are shown in Figs. 7 and 8, respectively. It is easily seen that the EDGES anomaly can be explained by powers $\omega _{max}< 0.544$ and $\delta _{max}< -0.05$. We also notice that we can only set an upper bound to the powers $\omega$ and $\delta$, since the electroweak length scale $\lambda _{EW}$ is an upper bound for the new length scale. Second, the stringent values for $\omega$ and $\delta$, to resolve the EDGES anomaly, with their respective errors will be available in the future, when the true new length scale will be estimated and known with higher energy accelerators and astrophysical observations. Third, we also point out that power $\delta <0$, which means that the correction decreases with increasing *E* as also seen in case 1. It may be noted that negative $\delta$ and positive $\lambda '$ is equivalent to positive $\delta$ and negative $\lambda '$ to leading order.

## Conclusion

In this work, we have studied the possibility that MDRs can account for the recent results of the EDGES collaboration, which has discovered an anomalous absorption signal in the CMB radiation spectrum. This signal is larger by about a factor of 2 with respect to the expected value (assuming that the background is described by the $\Lambda$CDM model), i.e., the EDGES anomaly. In particular, we have shown that the most commonly considered MDRs, namely cases 1-3, lead to a modified thermal spectrum and to the subsequent estimation of the parameters $(\eta , \alpha _0, \sqrt{\beta _0}, \alpha , \lambda ')\left| _{z=z_E} \approx 10^{32}\right.$. Unfortunately, the parameter values at redshift $z=z_E$ are outside the bounds allowed by, e.g., the electroweak experiments, since $(\eta , \alpha _0, \sqrt{\beta _0}, \alpha , \lambda ')\left| _{z=z_E} \gg \lambda _{EW}/\ell _P=10^{17}\right.$. However, given the precision of the CMB temperature in the current epoch, $z=0$, we were able to constrain these parameters to an upper bound $(\eta , \alpha _0, \sqrt{\beta _0}, \alpha , \lambda ')\left| _{z=0}\right. <10^{28}$ to be consistent with the observed CMB black body spectrum. The estimation of the MDR parameters from the EDGES anomaly at $z=z_E$, the bound obtained from electroweak experiments at $z=0$ and the BBN bound at $z\approx 3\times 10^8$ suggest that the MDR parameters should be functions of redshift *z* and as such could explain the EDGES anomaly. We can assume that the evolution of MDR parameters with time in the current epoch is slow or nearly constant, since we observe the same physics in all observable astrophysical objects such as distant galaxies. However, the time evolution of MDR parameters could have been faster in the early stages of the Universe as the EDGES anomaly suggests.

There is also another way out! As seen in Figs. 6, 7 and 8 and explained there, letting the powers $\omega$, $\gamma$ and $\delta$ vary does also explain the anomaly for finite ranges of those powers. To precisely fit the EDGES experiment, and set $\eta , \alpha _0, \sqrt{\beta _0}, \alpha , \lambda '=10^{17}$, bounded by the electroweak scale, we have studied the possibility of treating the powers $\omega$, $\gamma$ and $\delta$ of the MDRs as free parameters and estimating upper bounds to their values. Similar results were found in Ref. [41]. However, MDRs with non-trivial power dependencies require further research to better understand their importance for QG theories.

The results in this work indicate that MDRs originating from existing theories and thought experiments with minimal measurable length can provide a mechanism which explains the EDGES anomaly only if the MDR parameters are increasing functions of redshift *z*. Also, if the true QG theory with minimum measurable length predicts non-trivial deformation parameters as obtained from Figs. 6, 7 and 8, then such a theory can also provide a viable mechanism to explain the EDGES anomaly as well. It will be interesting to study the consequences of such deformation parameters in various contexts, such as GRBs physics [19]. We hope to report on this in the future.

## Data Availability

This manuscript has no associated data or the data will not be deposited. [Authors’ comment: The work is theoretical and did not involve any data, except for the one measurement in Eq. (13), cited from [60], which was used to evaluate the parameters of the different models.]

## Appendix A: 21-cm cosmology

In this Appendix, we briefly recall the main features of the 21-cm cosmology. First, we note that the 21-cm line is associated with the relative orientation of electron and proton spins (anti-parallel for the singlet level with lower energy $E_{\uparrow \downarrow }$, parallel for the triplet level with higher energy $E_{\uparrow \uparrow }$). This gives rise to a hyperfine energy splitting between the two energy levels of the 1s ground state of the hydrogen atom. The corresponding energy gap $E_{21}\equiv E_{\uparrow \uparrow }-E_{\uparrow \downarrow }$, hence that of the absorbed or emitted photons, is given by $E_{21} = 5.87\,\mu \mathrm{eV}$, which corresponds to a wavelength $\lambda _{21}^{\mathrm{rest}}=21$ cm or frequency $\nu _{21}^{\mathrm{rest}} = 1420\,\mathrm{MHz}$. Due to this 21-cm transition, a neutral hydrogen at the recombination, with redshift $z \lesssim z_{\mathrm{rec}}$, can act as a detector of the background photons that have been produced at higher redshifts. In the $\Lambda$CDM model such a photon background is given by the thermal radiation of the CMB with temperature $T_{CMB}(z) = T_{CMB,0}\, (1+z)$, where $T_{CMB,0} = 2.725\,K \simeq 2.35 \times 10^{-4}\,\mathrm{eV}$.

The frequency of the 21-cm transition falls in the Rayleigh-Jeans tail, where the intensity $I_\nu \propto T$. To study absorption and emission of light, we can therefore use the integrated radiative transfer equation (in a rest frame) written in terms of temperature [53, 77]

A1 $$\begin{aligned} T_b(\tau _\nu )=T_S\left( 1-e^{-\tau _\nu }\right) +T_{\gamma }\,\,e^{-\tau _\nu }~, \end{aligned}$$

where $T_b(\nu )$ is the observed absolute brightness temperature, $T_S(z)$ the so-called *spin temperature* defined by the ratio of the population of the excited state $n_2$ with respect to the ground state states $n_1$,

A2 $$\begin{aligned} \frac{n_2}{n_1}(z) \equiv \frac{g_2}{g_1} e^{-\frac{E_{21}}{k_BT_S(z)}}~, \end{aligned}$$

(where $g_2/g_1 =3$ indicates the ratio of the statistical degeneracy factors of the two levels) and $\tau _\nu$ is the optical depth (of the hydrogen cloud in our case) defined as [50, 53]

A3 $$\begin{aligned} \tau _\nu= & {} \int \mathrm {d}s\, \sigma _{21}\left( 1-e^{-\frac{E_{21}}{k_BT_S}}\right) \phi (\nu )\,n_0\nonumber \\\approx & {} \sigma _{21}\left( \frac{h\nu }{k_BT_S}\right) \left( \frac{N_{\mathrm {HI}}}{4}\right) \phi (\nu )~. \end{aligned}$$

In the above, $\phi (\nu )$ is the line profile which, in general, is a Voigt function normalized as $\int \phi (\nu )\mathrm {d}\nu =1$, $\mathrm {d}s$ is the line element between the source and the observer, $n_0$ is the number density of neutral hydrogen, $N_{\mathrm {HI}}=\int \mathrm {d}s\,n_0$ is the column density of neutral hydrogen and $\sigma _{21}$ is the absorption cross-section for the transition. The latter is defined as [50]

A4 $$\begin{aligned} \sigma _{21}=\frac{3\,c^2A_{21}}{8\pi \nu ^2}~, \end{aligned}$$

where $A_{21}$ is the Einstein coefficient for spontaneous emission. A relevant quantity in context of the 21-cm cosmology is the brightness temperature (the 21-cm brightness temperature is expressed relatively to the photon background at redshift *z*) [50, 53, 77]

A5 $$\begin{aligned} T_{21}(z)\equiv & {} \delta T_b (z)=\frac{T_S(z)-T_{\gamma }(z)}{1+z}(1-e^{-\tau _\nu })\nonumber \\ {}\simeq & {} \frac{T_S(z)-T_{\gamma }(z)}{1+z}\tau _{\nu } \nonumber \\\simeq & {} 23\,\mathrm{mK} \, (1+\delta _B)\, x_{H_I}(z) \,\left( \frac{\Omega _B\,h^2}{0.02}\right) \, \nonumber \\&\times \left( \frac{0.15}{\Omega _{\mathrm{m}}\,h^2}\right) ^{1/2}\, \left( \frac{1+z}{10} \right) ^{1/2} \,\left[ 1 - \frac{T_\gamma (z)}{T_S(z)} \right] ,\nonumber \\ \end{aligned}$$

where $\Omega _B\,h^2 = 0.02226$ is the baryon abundance, $\Omega _{\mathrm{m}}\,h^2=0.1415$ matter abundances [78], $\delta _B$ the baryon overdensity, $x_{H_I}$ the fraction of neutral hydrogen, and $T_{\gamma }(z)$ the effective temperature (at frequency $\nu _{21}(z)(=\nu _{21}^{\mathrm{rest}}/(1+z))$) of the photon background radiation (in the $\Lambda$CDM model it does coincide with $T_{CMB}(z)$). The spin temperature $T_S$ is related to the kinetic temperature of the gas $T_{\mathrm{gas}}$ by

A6 $$\begin{aligned} 1- \frac{T_{\gamma }}{T_S} \simeq \frac{x_c + x_\alpha }{1 + x_c + x_\alpha }\, \left( 1- \frac{T_{\gamma }}{T_{\mathrm{gas}}}\right) . \end{aligned}$$

Here the coefficients $x_{c}$ and $x_{\alpha }$ describe the coupling between the hyperfine levels and the gas, characterized by the fact that for $x_\alpha + x_c \gg 1$ (limit of strong coupling) it follows $T_S = T_{\mathrm{gas}}$, while for $x_\alpha = x_c =0$ (no coupling), it follows $T_S = T_{\gamma }$, which means that there is no signal.

## Appendix B: Modification of Einstein coefficients

Considering a gas of hydrogen, we study the MDR modification of absorption, spontaneous emission, and induced emission which take place when there is background radiation, with specific frequency passing through the gas. This will in turn provide a mechanism to study the MDR modification to the optical depth $\tau _\nu$. In this Appendix we follow the procedure outlined in [79].

If there are $N_2$ atoms in a higher energy state, the atoms will spontaneously decay to a lower energy state and emit photons with a specific frequency $\nu =\Delta E/h$, where $\Delta E$ is the energy difference between the two states (in our case it will be energy $E_{21}$ of the hyperfine transition). The transition rate for spontaneous emission is

B1 $$\begin{aligned} W_{21}^s=A_{21}N_2~, \end{aligned}$$

where $A_{21}$ is the Einstein coefficient for spontaneous emission. Note that here 1 and 2 refer to the lower and higher energy states respectively, and not as a subscript for 21-cm.

On the other hand, if we have $N_1$ atoms in the lower energy state and subject them to radiation with thermal spectrum $\rho _{MDR}(\nu )=\rho (\nu )R$ and frequency $\nu$ (which is a frequency corresponding to the hyperfine energy splitting), the radiation will be absorbed, and the atoms will transition to the higher energy state. The transition rate for induced absorption is

B2 $$\begin{aligned} W_{12}=B_{12}N_1\rho (\nu )R~, \end{aligned}$$

where $B_{12}$ is the Einstein coefficient for induced absorption and *R* is the MDR modification of the thermal spectrum originating from Eq. (7).

There is also a third possibility, where we have $N_2$ atoms in the higher energy state and subject them to radiation with thermal spectrum $\rho _{MDR}(\nu )$ and frequency $\nu$, the radiation will induce emission of new photons, originating from the transition to a lower level and traveling in the same direction as the incident radiation. The transition rate for induced emission is

B3 $$\begin{aligned} W_{21}^i=B_{21}N_2\rho (\nu )R~, \end{aligned}$$

where $B_{21}$ is the Einstein coefficient for induced emission. The total transition rate for emission is the sum of the spontaneous and induced emission transition rates

B4 $$\begin{aligned} W_{21}=W_{21}^s+W_{21}^i=N_2\left( A_{21}+B_{21}\rho (\nu )R\right) ~. \end{aligned}$$

The principle of detailed balance tells us that in thermal equilibrium the emission and absorption rates are equal, i.e., $W_{21}=W_{12}$. Using this, as well as Eqs. (A2), (8) and $E=h\nu$, we obtain the ratio of the Einstein coefficients

B5 $$\begin{aligned} \frac{A_{21}}{B_{21}}=\frac{8\pi h\nu ^3}{c^3}R~, \end{aligned}$$

and the ratio of induced absorption and emission coefficients

B6 $$\begin{aligned} \frac{B_{12}}{B_{21}}=\frac{g_2}{g_1}~. \end{aligned}$$

From Eq. (B5) we see that while the ratio of the Einstein coefficients is modified, it does not tell us how the individual coefficients are modified. However, Eqs. (B1), (B2) and (B3) suggest that only the *B* coefficients are modified by *R* and the *A* coefficient remains unmodified, since

B7 $$\begin{aligned} B_{12}\propto B_{21}\propto \frac{1}{R}\,\,\,\,\,\,\,\,\,\,\mathrm {and}\,\,\,\,\,\,\,\,\,\,A_{21}\not \propto f(R)~. \end{aligned}$$

Next we show that the absorption cross-section $\sigma _{21}$ does not depend on *R* as well. The driving equation to study absorption and emission of light in a gas is the radiative transfer equation, written in the differential form

B8 $$\begin{aligned} \frac{\mathrm {d}I_\nu }{\mathrm {d}s}=-\,\alpha (\nu )\,I_{\nu }~, \end{aligned}$$

where we neglect the emission part (we only need information on absorption, since we are looking for the absorption cross-section), $I_\nu =c\rho (\nu )R=I_{0\nu }R$ is the spectral intensity, $I_{0\nu }$ is the unmodified spectral intensity and $\alpha (\nu )$ is the absorption coefficient, related to the absorption cross-section as

B9 $$\begin{aligned} \alpha (\nu )=n_1\,\sigma _{21}\,\phi (\nu )~. \end{aligned}$$

By plugging $I_\nu =I_{0\nu }R$ in Eq. (B8), we find that the same radiative transfer equation holds also for $I_{0\nu }$, since $R\ne f(s)$ and reads as

B10 $$\begin{aligned} \frac{\mathrm {d}I_{0\nu }}{\mathrm {d}s}=-\,\alpha (\nu )\,I_{0\nu }~. \end{aligned}$$

The power of the incident beam with frequencies between $\nu$ and $\nu +\mathrm {d}\nu$ is absorbed by $N_1$ atoms reads as

B11 $$\begin{aligned} -\Delta P=h\nu \, W_{12}\,\phi (\nu )\mathrm {d}\nu =h\nu B_{12}N_1\rho (\nu )\,R\,\phi (\nu )\mathrm {d}\nu ,\nonumber \\ \end{aligned}$$

where $h\nu$ is the energy of a photon with frequency $\nu$, $W_{12}$ is the absorption transition rate defined in Eq. (B2) and $\phi (\nu )$ is the line profile defined in Eq. (A3). Writing the number of atoms in the ground state as $N_1=n_1A\Delta s$, where the atoms are confined in a volume $A\Delta s$ and the thermal spectrum $\rho (\nu )$ in terms of spectral intensity $I_\nu$, we get

B12 $$\begin{aligned} -\Delta P=\frac{h\nu }{c}B_{12}\,n_1A\,\Delta s\,I_{0\nu } R\,\phi (\nu )\mathrm {d}\nu ~. \end{aligned}$$

By the definition of the spectral intensity, we know that $\frac{\Delta P}{A\mathrm {d}\nu \Delta s} \implies \frac{\mathrm {d}I_{0\nu }}{\mathrm {d}s}$. Therefore we can rewrite Eq. (B12) as

B13 $$\begin{aligned} \frac{\mathrm {d}I_{0\nu }}{\mathrm {d}s} =-\frac{h\nu }{c}n_1B_{12}\,R\,\phi (\nu )\,I_{0\nu }~. \end{aligned}$$

By comparing the above equation with Eqs. (B10) and (B9), we obtain the absorption cross-section

B14 $$\begin{aligned} \sigma _{21}=\frac{h\nu }{c}B_{12}\,R~. \end{aligned}$$

By using Eqs. (B6) and (B5) in the above, we see that the factor *R* cancels and we obtain the final expression for the absorption cross-section

B15 $$\begin{aligned} \sigma _{21}=\frac{3\,c^2A_{21}}{8\pi \nu ^2}~, \end{aligned}$$

which is exactly the same as Eq. (A4). We see that the above absorption cross-section does not depend on *R* and therefore, according to Appendix A, MDRs do not modify the optical depth $\tau _\nu$.

## Footnotes

1 We use this definition to obtain the standard thermal spectrum, as can be found in [47, 48]. This differs from the definition used in [46] by an unimportant factor of $2\pi \hbar$, which has no effect on our calculations or results.

2 The EDGES collaboration found an absorption profile approximately in the range $z = 15-20$, with the minimum at the redshift $z_E = 17.2$.
